# Intuitionistic Fuzzy Cycles and Intuitionistic Fuzzy Trees

**DOI:** 10.1155/2014/305836

**Published:** 2014-02-18

**Authors:** Muhammad Akram, N. O. Alshehri

**Affiliations:** ^1^Department of Mathematics, University of the Punjab, New Campus, P.O. Box No. 54590, Lahore, Pakistan; ^2^Department of Mathematics, Faculty of Sciences (Girls), King Abdulaziz University, Jeddah, Saudi Arabia

## Abstract

Connectivity has an important role in neural networks, computer network, and clustering. In the design of a network, it is important to analyze connections by the levels. The structural properties of intuitionistic fuzzy graphs provide a tool that allows for the solution of operations research problems. In this paper, we introduce various types of intuitionistic fuzzy bridges, intuitionistic fuzzy cut vertices, intuitionistic fuzzy cycles, and intuitionistic fuzzy trees in intuitionistic fuzzy graphs and investigate some of their interesting properties. Most of these various types are defined in terms of levels. We also describe comparison of these types.

## 1. Introduction

A graph theory has many applications in different areas of computer science including data mining, image segmentation, clustering, image capturing, and networking. For example, a data structure can be designed in the form of trees; modeling of network topologies can be done using graph concepts. The most important concept of graph coloring is utilized in resource allocation and scheduling. The concepts of paths, walks, and circuits in graph theory are used in traveling salesman problem, database design concepts, and resource networking. This leads to the development of new algorithms and new theorems that can be used in tremendous applications.

A notion having certain influence on graph theory is fuzzy set, which is introduced by Zadeh [1] in 1965. Fuzzy graph theory is finding an increasing number of applications in modeling real time systems where the level of information inherent in the system varies with different levels of precision. Fuzzy models are becoming useful because of their aim in reducing the differences between the traditional numerical models used in engineering and sciences and the symbolic models used in expert systems.

Kaufmann's initial definition of a fuzzy graph [2] was based on Zadeh's fuzzy relations [3]. Rosenfeld [4] introduced the fuzzy analogue of several basic graph-theoretic concepts including bridges, cut nodes, connectedness, trees, and cycles. Bhattacharya [5] gave some remarks on fuzzy graphs, and Sunitha and Vijayakumar [6] characterized fuzzy trees. Bhutani and Rosenfeld [7] introduced the concepts of strong arcs, fuzzy end nodes, and geodesics in fuzzy graphs, and types of arcs in a fuzzy graph are described in [8]. Atanassov [9] introduced the concept of intuitionistic fuzzy relations and intuitionistic fuzzy graphs. Parvathi et al. [10, 11] have studied intuitionistic fuzzy graphs and intuitionistic fuzzy shortest hyperpath in a network. Karunambigai et al. [12] have described arcs in intuitionistic fuzzy graphs. Akram et al. [13–15] have discussed many concepts, including strong intuitionistic fuzzy graphs, intuitionistic fuzzy hypergraphs, and metric aspects of intuitionistic fuzzy graphs. In this paper, we introduce various types of intuitionistic fuzzy bridges, intuitionistic fuzzy cut vertices, intuitionistic fuzzy cycles, and intuitionistic fuzzy trees in intuitionistic fuzzy graphs and investigate some of their interesting properties.

We have used standard definitions and terminologies in this paper. For other notations, terminologies, and applications not mentioned in the paper, the readers should refer to [3, 5, 7, 8, 10, 16–23].

## 2. Preliminaries

In this section, we review some elementary concepts whose understanding is necessary to fully benefit from this paper.

By a graph, we mean a pair G* = (*V*, *E*), where *V* is the set and *E* is a relation on *V*. The elements of *V* are vertices of G* and the elements of *E* are edges of G*. We write *xy* ∈ *E* to mean that (*x*, *y*) ∈ *E*, and if *e* = *xy* ∈ *E*, we say that *x* and *y* are *adjacent*. A *path* in a graph G* is an alternating sequence of vertices and edges *v*_0_, *e*_1_, *v*_1_, *e*_2_, …, *v*_*n*−1_, *e*_*n*_, and *v*_*n*_. The path graph with *n* vertices is denoted by *P*_*n*_. A path is sometime denoted by *P*_*n*_ : *v*_0_ *v*_1_ ⋯ *v*_*n*_  (*n* > 0). The *length* of a path *P*_*n*_ in G* is *n*. A path *P*_*n*_ : *v*_0_ *v*_1_ ⋯ *v*_*n*_ in G* is called a *cycle* if *v*_0_ = *v*_*n*_ and *n* ≥ 3. Note that path graph, *P*_*n*_, has *n* − 1 edges and can be obtained from cycle graph, *C*_*n*_, by removing any edge. An undirected graph G* is *connected* if there is a path between each pair of distinct vertices. A *block* is a maximal biconnected subgraph of a given graph *G*. An edge *e* in a connected graph *G* is a *bridge* (cut-edge or cut arc) if *G* − *e* is disconnected. A vertex *v* in a connected graph *G* is a *cut vertex* if *G* − *v* is disconnected. The graphs with exactly *n* − 1 bridges are exactly the trees, and the graphs in which every edge is a bridge are exactly the forests. A *tree* is a connected graph which contains no cycles.

**Proposition 1**

Let *G* be a graph with *n* vertices. Then the following statements are equivalent.

- *G* *is connected and contains no cycles.*
- *G is connected and has n* − 1* edges.*
- *G has n* − 1* edges and contains no cycles.*
- *G is connected and each edge is a bridge.*
- *Any two vertices of* *G are connected by exactly one path.*
- *G contains no cycles, but the addition of any new edge creates exactly one cycle.*

A *spanning tree* in a connected graph *G* is a subgraph of *G* that includes all the vertices of *G* and is also a tree. A *forest* is an undirected graph; all of its connected components are trees; in other words, the graph consists of a disjoint union of trees.

A *fuzzy subset μ* on a set *X* is a map *μ* : *X* → [0,1]. A *fuzzy binary relation ν* on *X* is a fuzzy subset *ν* on *X* × *X*. By a fuzzy relation *ν*, we mean a fuzzy binary relation given by *ν* : *X* × *X* → [0,1]. Let *ν*∘*ν* be a fuzzy set of *E*⊆*V* × *V* defined by *ν*∘*ν*(*x*, *y*) = sup⁡{min⁡{*ν*(*x*, *y*), *ν*(*y*, *z*)} | *z* ∈ *V*}. Then *ν*∘*ν* is called the composition of *ν* with itself. Since composition is associative, we get *ν* ^*k*^ = *ν* ^*k*−1^∘*ν* for *k* = 1, 2,3, …. Define the fuzzy subset *ν* ^*∞*^ of *V* × *V* by

(1) $$\begin{array}{c} \nu^{\infty }\left(x,y\right)=\sup\left\{\nu^{k}\left(x,y\right):k=1,2,\ldots \right\}. \end{array}$$

*ν* ^*∞*^(*x*, *y*) denotes the “strength of connectedness” between two nodes *x* and *y*. That is, *ν* ^*∞*^(*x*, *y*) is defined as the maximum of the strengths of all paths between *x* and *y*.

In 1995, Atanassov [16] introduced the concept of intuitionistic fuzzy sets as a generalization of fuzzy sets [1]. Atanassov added a new component (which determines the degree of nonmembership) in the definition of fuzzy set. The fuzzy sets give the degree of membership of an element in a given set (and the nonmembership degree equals one minus the degree of membership), while intuitionistic fuzzy sets give both a degree of membership and a degree of nonmembership which are more or less independent from each other; the only requirement is that the sum of these two degrees is not greater than 1.

An intuitionistic fuzzy set (IFS, for short) on a universe *X* is an object of the form

(2) $$\begin{array}{c} A=\left\{\langle x,\mu_{A}\left(x\right),\nu_{A}\left(x\right)\rangle \;|\;x\in X\right\}, \end{array}$$

where *μ*_*A*_(*x*)  (∈[0,1]) is called degree of membership of *x* in *A*, *ν*_*A*_(*x*)  (∈[0,1]) is called degree of nonmembership of *x* in *A*, and *μ*_*A*_, *ν*_*A*_ satisfies the following condition for all *x* ∈ *X*, *μ*_*A*_(*x*) + *ν*_*A*_(*x*) ≤ 1. In particular, we use 0_~_ and 1_~_ to denote the *intuitionistic fuzzy empty set* and the *intuitionistic fuzzy whole set* in a set *L* such that 0_~_(*x*) = (0,1) and 1_~_(*x*) = (1,0), for each *x* ∈ *X*, respectively. An intuitionistic fuzzy relation *R* = (*μ*_*R*_(*x*, *y*), *ν*_*R*_(*x*, *y*)) in a universe *X* × *Y* (*R*(*X* → *Y*), for short) is an intuitionistic fuzzy set of the form

(3) $$\begin{array}{c} R=\left\{\langle \left(x,y\right),\mu_{A}\left(x,y\right),\nu_{A}\left(x,y\right)\rangle \;|\;\left(x,y\right)\in X\times Y\right\}, \end{array}$$

where *μ*_*A*_ : *X* × *Y* → [0,1] and *ν*_*A*_ : *X* × *Y* → [0,1]. The intuitionistic fuzzy relation *R* satisfies *μ*_*R*_(*x*, *y*) + *ν*_*R*_(*x*, *y*) ≤ 1 for all *x*, *y* ∈ *X*. An intuitionistic fuzzy relation *R* on universe *X* is called *reflexive* if *R*(*x*, *x*) = (1, 0) for each *x* ∈ *X*. *R* is called *symmetric* if *R*(*x*, *y*) = *R*(*y*, *x*) for any *x*, *y* ∈ *X*.

**Definition 2**

The *height* of an intuitionistic fuzzy set *A* is defined as

(4) h(A)=sup⁡x∈X(A)(x)=(sup⁡x∈XμA(x),inf⁡x∈XνA(x))=(h(μA),h(νA)).

We will say that intuitionistic fuzzy set *A* is *normal* if there is at least one *x* ∈ *X* such that *μ*_*A*_(*x*) = 1. The *depth* of an intuitionistic fuzzy set *A* is defined as

(5) d(A)=inf⁡x∈X(A)(x)=(inf⁡x∈XμA(x),sup⁡x∈XνA(x))=(d(μA),d(νA)).

*Notation.* (1) Let 0 = (0,0); then (*s*, *t*)∈(0, *h*(*A*)] means that (*s*, *t*)∈(0, *h*(*μ*_*A*_)]×(0, *h*(*ν*_*A*_)].

(2) (*s*, *t*)∈(*d*(*A*), *h*(*A*)] means that (*s*, *t*)∈(*d*(*μ*_*A*_), *h*(*μ*_*A*_)]×(*d*(*ν*_*A*_), *h*(*ν*_*A*_)].

**Definition 3 (see [14])**

By an *intuitionistic fuzzy graph* (IFG), one means a pair *G* = (*A*, *B*) in which *A* = (*μ*_*A*_, *ν*_*A*_) is an intuitionistic fuzzy set on *V* and *B* = (*μ*_*B*_, *ν*_*B*_) is an intuitionistic fuzzy relation on *E* such that *μ*_*B*_(*x*, *y*) ≤ min⁡(*μ*_*A*_(*x*), *μ*_*A*_(*y*)), *ν*_*B*_(*x*, *y*) ≥ max⁡(*ν*_*A*_(*x*), *ν*_*A*_(*y*)), and 0 ≤ *μ*_*B*_(*x*, *y*) + *ν*_*B*_(*x*, *y*) ≤ 1 for all (*x*, *y*) ∈ *E*. Note that *B* is a symmetric intuitionistic fuzzy relation on *A*.

**Definition 4 (see [12, 14])**

An intuitionistic fuzzy graph is called *complete* if *μ*_*B*_(*x*, *y*) = min⁡(*μ*_*A*_(*x*), *μ*_*A*_(*y*)) and *ν*_*B*_(*x*, *y*) = max⁡(*ν*_*A*_(*x*), *ν*_*A*_(*y*)) for all *x*, *y*.

**Definition 5 (see [9])**

The support of *A* is defined by

(6) A∗=(μA∗,νA∗)={x∈V ∣ μA(x)>0, νA(x)>0}.

The *support* of *B* is defined by

(7) B∗=(μB∗,νB∗)={(x,y)∈E ∣ μB(x,y)>0, νB(x,y)>0}.

Let G* = (A*, B*). For *s*, *t* ∈ [0,1], *A* ^(*s*,*t*)^ = {*x* ∈ *V* | *μ*_*A*_(*x*) ≥ *s*,  *ν*_*A*_(*x*) ≤ *t*} is called an (*s*, *t*)*-level subset* of *A* and *B* ^(*s*,*t*)^ = {(*x*, *y*) ∈ *E* | *μ*_*B*_(*x*, *y*) ≥ *s*,  *ν*_*B*_(*x*, *y*) ≤ *t*} is called an (*s*, *t*)*-level subset* of *B*. Let *G* ^(*s*,*t*)^ = (*A* ^(*s*,*t*)^, *B* ^(*s*,*t*)^).

**Definition 6 (see [12])**

A path *P* in a intuitionistic fuzzy graph *G* is an sequence of distinct vertices *v*_1_, *v*_2_,…, *v*_*n*_ such that either one of the following condition is satisfied:

1. *μ*_*B*_(*x*, *y*) > 0 and *ν*_*B*_(*x*, *y*) > 0 for some *x*, *y*;
2. *μ*_*B*_(*x*, *y*) > 0 and *ν*_*B*_(*x*, *y*) = 0 for some *x*, *y*;
3. *μ*_*B*_(*x*, *y*) = 0 and *ν*_*B*_(*x*, *y*) > 0 for some *x*, *y*.

When *μ*_*B*_(*x*, *y*) = *ν*_*B*_(*x*, *y*) = 0 for some *x*, *y*, there is no edge between *x* and *y*. Otherwise, there exists an edge between *x* and *y*.

**Definition 7 (see [12])**

An intuitionistic fuzzy graph *G* is *connected* if any two vertices are joined by a path.

**Definition 8 (see [12])**

If *x*, *y* ∈ *V*, the *μ*-*strength of connectedness* between *x* and *y* is

(8) μB∞(x,y)=sup⁡{μBk(x,y) ∣ k=1,2,…,n},μB∞(x,y) =sup⁡⁡{μB(x,v1)∧μB(v1,v2)∧⋯∧μB(vk−1,y) ∣  x,v1,v2,…,vk−1,y∈V, k=1,2,…,n}.

The *ν*-strength of connectedness between *x* and *y* is

(9) νB∞(x,y)=inf⁡{νBk(x,y) ∣ k=1,2,…,n},νB∞(x,y)=inf⁡⁡{νB(x,v1)∨νB(v1,v2)∨⋯∨νB(vk−1,y) ∣  x,v1,v2,…,vk−1,y∈V, k=1,2,…,n}.

The *μ*-strength and *ν*-strength of connectedness between *x* and *y* in *G* are denoted by *μ*_*G*_^*∞*^(*x*, *y*) and *ν*_*G*_^*∞*^(*x*, *y*), respectively. Also *μ*_*B*_^′*∞*^(*x*, *y*) and *ν*_*B*_^′*∞*^(*x*, *y*) denote *μ*_*G*−(*x*,*y*)_^*∞*^(*x*, *y*) and *ν*_*G*−(*x*,*y*)_^*∞*^(*x*, *y*), where *G* − (*x*, *y*) is obtained from *G* by deleting the arc (*x*, *y*).

## 3. Bridges, Cut Vertices, and Blocks

Though the concept of path and connectedness in intuitionistic fuzzy graph is analogous to crisp graph, the other concepts like intuitionistic fuzzy tree and intuitionistic fuzzy bridge differ from those in crisp graph. In crisp graph, a cut node is the one whose removal from the graph disconnects the graph. A cut edge or bridge is also an edge whose removal disconnects the graph. But in intuitionistic fuzzy graph, the definitions of intuitionistic fuzzy bridge and intuitionistic fuzzy cut node are not so.

**Definition 9 (see [12])**

A bridge (*x*, *y*) in *G* is said to be *μ*-bridge, if deleting (*x*, *y*) reduces the *μ*-strength of connectedness between some pair of vertices. A bridge (*x*, *y*) is said to be *ν*-*bridge* if deleting (*x*, *y*) increases the *ν*-strength of connectedness between some pair of vertices. A bridge (*x*, *y*) is said to be an intuitionistic fuzzy bridge if it is *μ*-bridge and *ν*-bridge.

**Definition 10**

Let (*x*, *y*) ∈ *E*.

1. (*x*, *y*) is called a *bridge* if (*x*, *y*) is a bridge of G* = (A*, B*).
2. (*x*, *y*) is called an *intuitionistic fuzzy bridge* if ${\overset{´}{\mu }}_{B}^{\infty }(u,v)<\mu_{B}^{\infty }(u,v)$ and ${\overset{´}{\nu }}_{B}^{\infty }(u,v)>\nu_{B}^{\infty }(u,v)$ for some (*u*, *v*) ∈ *E*, where ${\overset{´}{\mu }}_{B}^{}$ and ${\overset{´}{\nu }}_{B}^{}$ are *μ*_*B*_ and *ν*_*B*_ restricted to *V* × *V* − {(*x*, *y*), (*y*, *x*)}.
3. (*x*, *y*) is called a *weak intuitionistic fuzzy bridge* if there exists (*s*, *t*)∈(0, *h*(*B*)] such that (*x*, *y*) is a bridge of *G* ^(*s*,*t*)^.
4. (*x*, *y*) is called a *partial intuitionistic fuzzy bridge* if (*x*, *y*) is a bridge for *G* ^(*s*,*t*)^ for all (*s*, *t*)∈(*d*(*B*), *h*(*B*)]∪{*h*(*B*)}.
5. (*x*, *y*) is called a full intuitionistic fuzzy bridge if (*x*, *y*) is a bridge for *G* ^(*s*,*t*)^ for all (*s*, *t*)∈(0, *h*(*B*)].

**Example 11**

**Figure 1:**
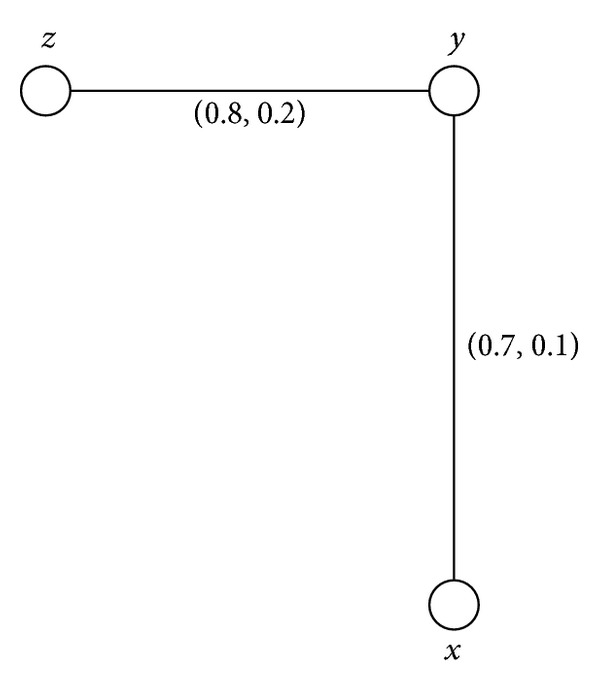
Connected intuitionistic fuzzy graph.

Consider a connected intuitionistic fuzzy graph as shown in Figure 1.

By routine computations, we have *d*(*B*) = (0.7,0.2) and *h*(*B*) = (0.8,0.1). Thus (*s*, *t*)∈(0, *h*(*B*)] means that (*s*, *t*)∈(0,0.8]×(0,0.1]. For 0 < *s* ≤ 0.7, 0 < *t* ≤ 0.2, *G* ^(*s*,*t*)^ = (*V*, {(*x*, *y*), (*y*, *z*)}). For 0.7 < *s* ≤ 0.8, 0 < *t* ≤ 0.1, *G* ^(*s*,*t*)^ = (*V*, {(*y*, *z*)}). Hence we conclude that (*y*, *z*) is a full intuitionistic fuzzy bridge and (*x*, *y*) is a weak intuitionistic fuzzy bridge but not a partial intuitionistic fuzzy bridge. Both (*x*, *y*) and (*y*, *z*) are bridges and intuitionistic fuzzy bridges.

**Example 12**

**Figure 2:**
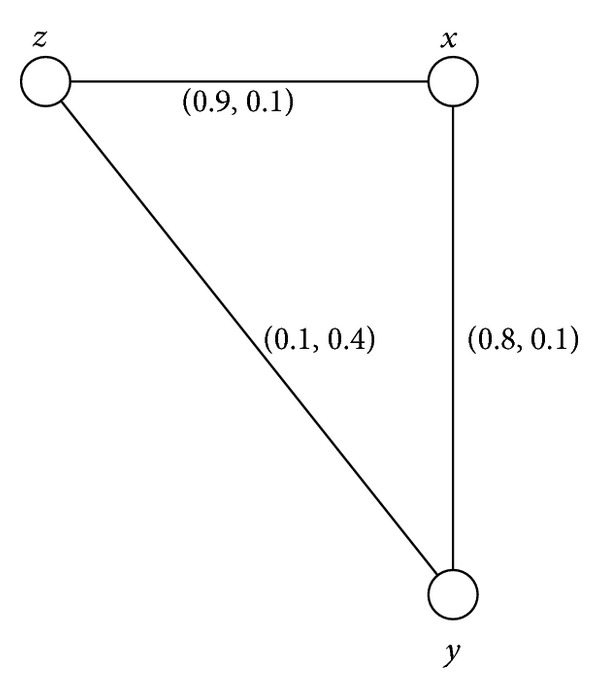
Connected intuitionistic fuzzy graph.

Consider a connected intuitionistic fuzzy graph as shown in Figure 2.

By routine computations, we have *d*(*B*) = (0.1,0.4) and *h*(*B*) = (0.9,0.1). For 0 < *s* ≤ 0.1, 0 < *t* ≤ 0.4, *G* ^(*s*,*t*)^ = (*V*, {(*x*, *y*), (*x*, *z*), (*y*, *z*)}). For 0.1 < *s* ≤ 0.8, 0 < *t* ≤ 0.1, *G* ^(*s*,*t*)^ = (*V*, {(*x*, *y*), (*x*, *z*)}). For 0.8 < *s* ≤ 0.9, 0 < *t* ≤ 0.1, *G* ^(*s*,*t*)^ = (*V*, {(*x*, *z*)}). Thus (*x*, *z*) is an intuitionistic fuzzy bridge and a partial intuitionistic fuzzy bridge but not a bridge. The edge (*y*, *z*) is not any of five types of bridges.

**Example 13**

Consider a connected graph G* = (*V*, *E*) such that *V* = {*x*, *y*, *z*} and *E* = {(*x*, *y*), (*y*, *z*), (*x*, *z*)}. Let *A* be an intuitionistic fuzzy set of *V* and let *B* be an intuitionistic fuzzy set of *E*⊆*V* × *V* defined by

(10) μA(x)=μA(y)=μA(z)=1,νA(x)=νA(y)=νA(z)=0,μB(x,y)=μB(y,z)=μB(x,z)=0.9,νB(x,y)=νB(y,z)=νB(x,z)=0.1.

Routine computations show that connected intuitionistic fuzzy graph *G* has no bridges of any of the five types.

**Example 14**

Consider a connected graph G* = (*V*, *E*) such that *V* = {*x*, *y*, *z*, *w*} and *E* = {(*x*, *y*), (*y*, *z*), (*x*, *z*), (*z*, *w*)}. Let *A* be an intuitionistic fuzzy set of *V* and let *B* be an intuitionistic fuzzy set of *E*⊆*V* × *V* defined by

(11) $$\begin{array}{c} \mu_{A}\left(x\right)=\mu_{A}\left(y\right)=\mu_{A}\left(z\right)=\mu_{A}\left(w\right)=1,\\ \nu_{A}\left(x\right)=\nu_{A}\left(y\right)=\nu_{A}\left(z\right)=\nu_{A}\left(w\right)=0,\\ \mu_{B}\left(x,y\right)=\mu_{B}\left(y,z\right)=0.1,\\ \mu_{B}\left(x,z\right)=\mu_{B}\left(w,z\right)=0.9,\\ \nu_{B}\left(x,y\right)=\nu_{B}\left(y,z\right)=0.5,\\ \nu_{B}\left(x,z\right)=\nu_{B}\left(w,z\right)=0.1. \end{array}$$

By routine computations, we have *d*(*B*) = (0.1,0.5) and *h*(*B*) = (0.9,0.1). For 0 < *s* ≤ 0.1, 0 < *t* ≤ 0.5, *G* ^(*s*,*t*)^ = (*V*, {(*x*, *y*), (*y*, *z*), (*x*, *z*), (*z*, *w*)}). For 0.1 < *s* ≤ 0.9, 0 < *t* ≤ 0.1, *G* ^(*s*,*t*)^ = (*V*, {(*x*, *z*), (*z*, *w*)}). Thus (*z*, *w*) is a full intuitionistic fuzzy bridge and (*x*, *z*) is a partial intuitionistic fuzzy bridge but not a full intuitionistic fuzzy bridge.

We state the following propositions without their proofs.

**Proposition 15**

Let (*x*, *y*) be a bridge in G*. Then (*x*, *y*) is an intuitionistic fuzzy bridge if and only if $\mu_{B}(x,y)>{\overset{´}{\mu }}_{B}^{\infty }(x,y)$ and $\nu_{B}(x,y)<{\overset{´}{\nu }}_{B}^{\infty }(x,y)$.

**Proposition 16**

(*x*, *y*) is an intuitionistic fuzzy bridge if and only if (*x*, *y*) is not the weakest bridge of any cycle.

**Proposition 17**

(*x*, *y*) is an intuitionistic fuzzy bridge if and only if (*x*, *y*) is a bridge for G* and *μ*_*B*_(*x*, *y*) = *h*(*μ*_*B*_) and *ν*_*B*_(*x*, *y*) = *h*(*ν*_*B*_).

**Proof**

Suppose that (*x*, *y*) is a full bridge. Then (*x*, *y*) is a bridge for *G* ^(*s*,*t*)^ for all (*s*, *t*)∈(0, *h*(*B*)] = (0, *h*(*μ*_*B*_)]×(0, *h*(*ν*_*B*_)]. Hence (*x*, *y*)∈*B* ^*h*(*B*)^ and so *μ*_*B*_(*x*, *y*) = *h*(*μ*_*B*_) and *ν*_*B*_(*x*, *y*) = *h*(*ν*_*B*_). Since (*x*, *y*) is a bridge for *G* ^(*s*,*t*)^ for all (*s*, *t*)∈(0, *h*(*B*)] = (0, *h*(*μ*_*B*_)]×(0, *h*(*ν*_*B*_)], it follows that (*x*, *y*) is a bridge for G* since *V* = *A* ^*d*(*B*)^ and *E* = *B* ^*h*(*B*)^.

Conversely, suppose that (*x*, *y*) is a bridge for G* and *μ*_*B*_(*x*, *y*) = *h*(*μ*_*B*_) and *ν*_*B*_(*x*, *y*) = *h*(*ν*_*B*_). Then (*x*, *y*) ∈ *B* ^(*s*,*t*)^ for all (*s*, *t*)∈(0, *h*(*B*)]. Thus since also (*x*, *y*) is a bridge for G*, (*x*, *y*) is a bridge for *G* ^(*s*,*t*)^ for all (*s*, *t*)∈(0, *h*(*B*)] since each *G* ^(*s*,*t*)^ is a subgraph of G*. Hence (*x*, *y*) is a full intuitionistic fuzzy bridge.

**Proposition 18**

Suppose that (*x*, *y*) is not contained in a cycle of G*. Then the following conditions are equivalent:

1. *μ*_*B*_(*x*, *y*) = *h*(*μ*_*B*_)* and  ν*_*B*_(*x*, *y*) = *h*(*ν*_*B*_)*;*
2. (*x*, *y*)* is a partial intuitionistic fuzzy bridge;*
3. (*x*, *y*)* is an intuitionistic full fuzzy bridge.*

**Proof**

Since (*x*, *y*) is not contained in a cycle of G*, (*x*, *y*) is a bridge of G*. Hence by Proposition 17, (1) ⇔(3). Clearly, (3) ⇔(2). Suppose that (2) holds. Then (*x*, *y*) is a bridge for *G* ^(*s*,*t*)^ for all (*s*, *t*)∈(*d*(*B*), *h*(*B*)] and so (*x*, *y*) ∈ *B* ^*h*(*B*)^. Thus *μ*_*B*_(*x*, *y*) = *h*(*μ*_*B*_) and *ν*_*B*_(*x*, *y*) = *h*(*ν*_*B*_); that is, (1) holds.

**Proposition 19**

If (*x*, *y*) is a bridge, then (*x*, *y*) is a weak intuitionistic fuzzy bridge and an intuitionistic fuzzy bridge.

**Proposition 20**

(*x*, *y*) is an intuitionistic fuzzy bridge if and only if (*x*, *y*) is a weak bridge.

**Proof**

Suppose that (*x*, *y*) is a weak intuitionistic fuzzy bridge. Then ∃(*s*, *t*)∈(0, *h*(*B*)] such that (*x*, *y*) is a bridge for *G* ^(*s*,*t*)^. Hence removal of (*x*, *y*) disconnects *G* ^(*s*,*t*)^. Thus any path from *x* to *y* in *G* has an edge (*u*, *v*) with *μ*_*B*_(*u*, *v*) < *s*, *ν*_*B*_(*u*, *v*) > *t*. Thus the removal of (*x*, *y*) results in *μ*_*B*_^′*∞*^(*x*, *y*) < *s* ⩽ *μ* ^*∞*^(*x*, *y*), *ν*_*B*_^′*∞*^(*x*, *y*) < *t* ⩽ *ν* ^*∞*^(*x*, *y*). Hence (*x*, *y*) is an intuitionistic fuzzy bridge.

Conversely, suppose that (*x*, *y*) is an intuitionistic fuzzy bridge. Then ∃(*u*, *v*) such that removal of (*x*, *y*) results in *μ*_*B*_^′*∞*^(*u*, *v*) < *μ*_*B*_^*∞*^(*u*, *v*), *ν*_*B*_^′*∞*^(*u*, *v*) > *ν*_*B*_^*∞*^(*u*, *v*). Hence (*x*, *y*) is on every strongest path connecting *u* and *v* and in fact *μ*_*B*_(*u*, *v*)⩾ and *ν*_*B*_(*u*, *v*)⩽ this value. Thus there does not exist a path (other than (*x*, *y*)) connecting *x* and *y* in *G* ^(*μ*_*B*_(*x*,*y*),*ν*_*B*_(*x*,*y*))^, else this other path without (*x*, *y*) would be of strength ⩾*μ*_*B*_(*x*, *y*) and ⩽*ν*_*B*_(*x*, *y*) and would be part of a path connecting *u* and *v* of strongest length, contrary to the fact that (*x*, *y*) is on every such path. Hence (*x*, *y*) is a bridge of *G* ^(*μ*_*B*_(*x*,*y*),*ν*_*B*_(*x*,*y*))^ and 0 < *μ*_*B*_(*x*, *y*) ≤ *h*(*μ*_*B*_), 0 < *ν*_*B*_(*x*, *y*) ≤ *h*(*ν*_*B*_). Thus *μ*_*B*_(*x*, *y*) and *ν*_*B*_(*x*, *y*) are desired (*s*, *t*).

**Definition 21 (see [12])**

A vertex *x* ∈ *V* in *G* is called *μ*-*cut vertex* if deleting it reduces the *μ*-strength of connectedness between some pairs of vertices. A vertex *x* ∈ *V* is called *ν*-*cut vertex* if deleting it increases the *ν*-strength of connectedness between some pairs of vertices. A vertex *x* ∈ *V* is an intuitionistic fuzzy cut vertex if it is *μ*-cut vertex and *ν*-cut vertex.

**Definition 22**

Let *x* ∈ *V*.

1. *x* is called a *cut vertex* if *x* is a cut-vertex of G* = (A*, B*).
2. *x* is called an *intuitionistic fuzzy cut-vertex* if *μ*_*B*_^′*∞*^(*u*, *v*) < *μ*_*B*_^*∞*^(*u*, *v*) and *ν*_*B*_^′*∞*^(*u*, *v*) > *ν*_*B*_^*∞*^(*u*, *v*) for some *u*, *v* ∈ *V*, where *μ*_*B*_′ and *ν*_*B*_′ are *μ*_*B*_ and *ν*_*B*_ restricted to *V* × *V* − {(*x*, *z*), (*z*, *x*) | *z* ∈ *V*}.
3. *x* is called a *weak intuitionistic fuzzy cut-vertex* if there exists (*s*, *t*)∈(0, *h*(*B*)] such that *x* is a cut-vertex of *G* ^(*s*,*t*)^.
4. *x* is called a *partial intuitionistic fuzzy cut-vertex* if *x* is a cut-vertex for *G* ^(*s*,*t*)^ for all (*s*, *t*)∈(*d*(*B*), *h*(*B*)]∪{*h*(*B*)}.
5. *x* is called a *full intuitionistic fuzzy cut-vertex* if *x* is a cut-vertex for *G* ^(*s*,*t*)^ for all (*s*, *t*)∈(0, *h*(*B*)].

**Example 23**

**Figure 3:**
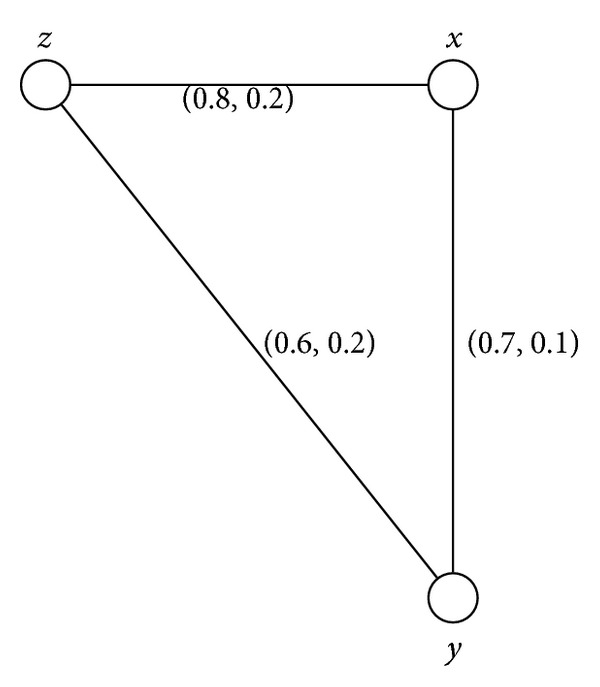
Connected intuitionistic fuzzy graph.

Consider a connected intuitionistic fuzzy graph as shown in Figure 3.

By routine computations, we have *d*(*B*) = (0.6,0.2), *h*(*B*) = (0.8,0.1). Thus (*s*, *t*)∈(0, *h*(*B*)] means that (*s*, *t*)∈(0,0.8]×(0,0.1]. For 0 < *s* ≤ 0.6 and 0 < *t* ≤ 0.2, *G* ^(*s*,*t*)^ = (*V*, {(*x*, *y*), (*y*, *z*), (*x*, *z*)}). For 0.6 < *s* ≤ 0.7 and 0 < *t* ≤ 0.2, *G* ^(*s*,*t*)^ = (*V*, {(*x*, *y*), (*x*, *z*)}). For 0.6 < *s* ≤ 0.8 and 0 < *t* ≤ 0.1, *G* ^(*s*,*t*)^ = (*V*, {(*x*, *z*)}). Thus *x* is an intuitionistic fuzzy cut-vertex and a weak intuitionistic fuzzy cut-vertex but neither a cut-vertex nor a partial cut-vertex.

**Example 24**

Consider a connected graph G* = (*V*, *E*) such that *V* = {*x*, *y*, *z*} and *E* = {(*x*, *y*), (*y*, *z*), (*x*, *z*)}. Let *A* be an intuitionistic fuzzy set of *V* and let *B* be an intuitionistic fuzzy set of *E*⊆*V* × *V* defined by

(12) $$\begin{array}{c} \mu_{A}\left(x\right)=\mu_{A}\left(y\right)=\mu_{A}\left(z\right)=1,\\ \nu_{A}\left(x\right)=\nu_{A}\left(y\right)=\nu_{A}\left(z\right)=0,\\ \mu_{B}\left(x,y\right)=\mu_{B}\left(x,z\right)=0.9,\\ \mu_{B}\left(y,z\right)=0.5,\\ \nu_{B}\left(x,y\right)=\nu_{B}\left(x,z\right)=0.1,\\ \nu_{B}\left(y,z\right)=0.4. \end{array}$$

By routine computations, we have *d*(*B*) = (0.5,0.4) and *h*(*B*) = (0.9,0.1). For 0 < *s* ≤ 0.5 and 0 < *t* ≤ 0.4, *G* ^(*s*,*t*)^ = (*V*, {(*x*, *y*), (*y*, *z*), (*x*, *z*)}). For 0.5 < *s* ≤ 0.9 and 0 < *t* ≤ 0.1, *G* ^(*s*,*t*)^ = (*V*, {(*x*, *y*), (*x*, *z*)}). Thus *x* is an intuitionistic fuzzy cut-vertex and a partial intuitionistic fuzzy cut-vertex but neither a cut-vertex nor a full cut-vertex.

**Example 25**

Consider a connected graph G* = (*V*, *E*) such that *V* = {*x*, *y*, *z*} and *E* = {(*x*, *y*), (*y*, *z*), (*x*, *z*)}. Let *A* be an intuitionistic fuzzy set of *V* and let *B* be an intuitionistic fuzzy set of *E*⊆*V* × *V* defined by

(13) $$\begin{array}{c} \mu_{A}\left(x\right)=\mu_{A}\left(y\right)=\mu_{A}\left(z\right)=1,\\ \nu_{A}\left(x\right)=\nu_{A}\left(y\right)=\nu_{A}\left(z\right)=0,\\ \mu_{B}\left(x,y\right)=\mu_{B}\left(x,z\right)=0.9,\\ \nu_{B}\left(x,y\right)=\nu_{B}\left(x,z\right)=0.1. \end{array}$$

By routine computations, we have *d*(*B*) = (0.9,0.1) and *h*(*B*) = (0.9,0.1). For 0 < *s* ≤ 0.9 and 0 < *t* ≤ 0.1, *G* ^(*s*,*t*)^ = (*V*, {(*x*, *y*), (*x*, *z*)}). Thus *x* is a full intuitionistic fuzzy cut-vertex, an intuitionistic fuzzy cut-vertex, and a cut-vertex.

We state the following propositions without their proofs.

**Proposition 26**

Let *G* be an intuitionistic fuzzy graph such that G* is a cycle. Then a node is an intuitionistic fuzzy cut node of *G* if and only if it is a common node of two intuitionistic fuzzy bridges.

**Proposition 27**

If *z* is a common node of at least two intuitionistic fuzzy bridges, then *z* is an intuitionistic fuzzy cut node.

**Proposition 28**

If *G* is a complete intuitionistic fuzzy graph, then *μ*_*B*_^*∞*^(*u*, *v*) = *μ*_*B*_(*u*, *v*) and *ν*_*B*_^*∞*^(*u*, *v*) = *ν*_*B*_(*u*, *v*).

**Proposition 29**

A complete intuitionistic fuzzy graph has no intuitionistic fuzzy cut vertex.

**Definition 30**

(1) *G* is called a *block* if G* is a block.

(2) *G* is called an *intuitionistic fuzzy block* if it has no intuitionistic fuzzy cut vertices.

(3) *G* is called a *weak intuitionistic fuzzy block* if there exists (*s*, *t*)∈(0, *h*(*B*)] such that *G* ^(*s*,*t*)^ is a block.

(4) *G* is called a *partial intuitionistic fuzzy block* if *G* ^(*s*,*t*)^ is a block for all (*s*, *t*)∈(*d*(*B*), *h*(*B*)]∪{*h*(*B*)}.

(5) *G* is called a *full intuitionistic fuzzy block* if *G* ^(*s*,*t*)^ is a block for all (*s*, *t*)∈(0, *h*(*B*)].

**Example 31**

**Figure 4:**
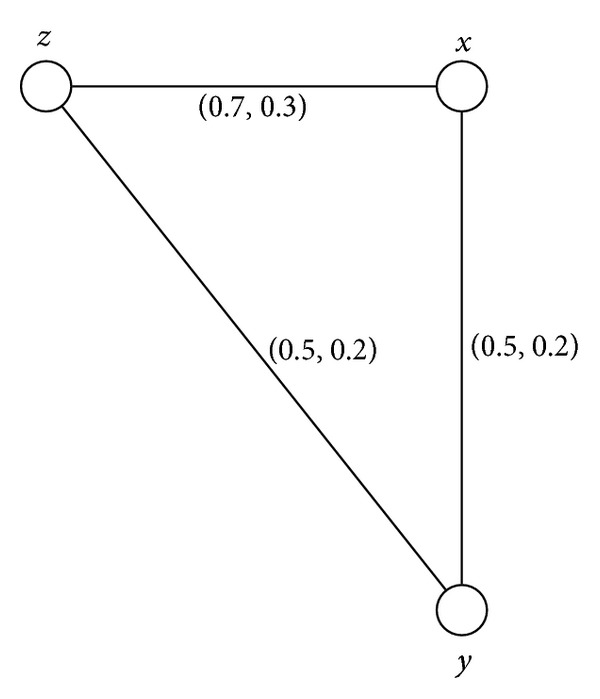
Connected intuitionistic fuzzy graph.

Consider a connected intuitionistic fuzzy graph as shown in Figure 4.

By routine computations, we have *d*(*B*) = (0.5,0.3) and *h*(*B*) = (0.7,0.2). Thus (*s*, *t*)∈(0, *h*(*B*)] means that (*s*, *t*)∈(0,0.7]×(0,0.2]. For 0 < *s* ≤ 0.5 and 0 < *t* ≤ 0.3, *G* ^(*s*,*t*)^ = (*V*, {(*x*, *y*), (*y*, *z*), (*x*, *z*)}). For 0.5 < *s* ≤ 0.7 and 0 < *t* ≤ 0.2, *G* ^(*s*,*t*)^ = (*V*, {(*x*, *z*)}). Thus *G* is a block, an intuitionistic fuzzy block, and a weak intuitionistic fuzzy block. *G* is not a partial intuitionistic fuzzy block since *G* ^(*s*,*t*)^ is not a block for 0.5 < *s* ≤ 0.7, 0 < *t* ≤ 0.2.

**Example 32**

Consider a connected graph G* = (*V*, *E*) such that *V* = {*x*, *y*, *z*} and *E* = {(*x*, *y*), (*y*, *z*), (*x*, *z*)}. Let *A* be an intuitionistic fuzzy set of *V* and let *B* be an intuitionistic fuzzy set of *E*⊆*V* × *V* defined by

(14) $$\begin{array}{c} \mu_{A}\left(x\right)=\mu_{A}\left(y\right)=\mu_{A}\left(z\right)=1,\\ \nu_{A}\left(x\right)=\nu_{A}\left(y\right)=\nu_{A}\left(z\right)=0,\\ \mu_{B}\left(x,y\right)=\mu_{B}\left(x,z\right)=0.9,\\ \mu_{B}\left(y,z\right)=0.5,\\ \nu_{B}\left(x,y\right)=\nu_{B}\left(x,z\right)=0.1,\\ \nu_{B}\left(y,z\right)=0.4. \end{array}$$

By routine computations, we have *d*(*B*) = (0.5,0.4) and *h*(*B*) = (0.9,0.1). For 0 < *s* ≤ 0.5 and 0 < *t* ≤ 0.4, *G* ^(*s*,*t*)^ = (*V*, {(*x*, *y*), (*y*, *z*), (*x*, *z*)}). For 0.5 < *s* ≤ 0.9 and 0 < *t* ≤ 0.1, *G* ^(*s*,*t*)^ = (*V*, {(*x*, *y*), (*x*, *z*)}). Thus *G* is a block and a weak intuitionistic fuzzy block. However, *G* is not an intuitionistic fuzzy block since *x* is an intuitionistic fuzzy cut vertex of *G*. Also *G* is not a partial intuitionistic fuzzy block since *x* is a cut vertex for 0.5 < *s* ≤ 0.9 and 0 < *t* ≤ 0.1.

**Example 33**

Consider a connected graph G* = (*V*, *E*) such that *V* = {*x*, *y*, *z*} and *E* = {(*x*, *y*), (*y*, *z*), (*x*, *z*)}. Let *A* be an intuitionistic fuzzy set of *V* and let *B* be an intuitionistic fuzzy set of *E*⊆*V* × *V* defined by

(15) $$\begin{array}{c} \mu_{A}\left(x\right)=\mu_{A}\left(y\right)=\mu_{A}\left(z\right)=1,\\ \nu_{A}\left(x\right)=\nu_{A}\left(y\right)=\nu_{A}\left(z\right)=0,\\ \mu_{B}\left(x,y\right)=\mu_{B}\left(x,z\right)=\mu_{B}\left(y,z\right)=0.9,\\ \nu_{B}\left(x,y\right)=\nu_{B}\left(x,z\right)=\nu_{B}\left(y,z\right)=0.1. \end{array}$$

By routine computations, we have *d*(*B*) = (0.9,0.1) and *h*(*B*) = (0.9,0.1). For 0 < *s* ≤ 0.9 and 0 < *t* ≤ 0.1, *G* ^(*s*,*t*)^ = (*V*, {(*x*, *y*), (*y*, *z*), (*x*, *z*)}). Thus *G* is a block, an intuitionistic fuzzy block, and a full intuitionistic fuzzy block.

**Definition 34**

A connected intuitionistic fuzzy graph *G* is said to be *firm* if

(16) $$\begin{array}{c} \min\left\{\mu_{A}\left(x\right)\;|\;x\in V\right\}\geq \max\left\{\mu_{B}\left(x,y\right)\;|\;\left(x,y\right)\in E\right\},\\ \max\left\{\nu_{A}\left(x\right)\;|\;x\in V\right\}\leq \min\left\{\nu_{B}\left(x,y\right)\;|\;\left(x,y\right)\in E\right\}. \end{array}$$

**Example 35**

All connected intuitionistic fuzzy graphs as shown in Figures 1, 2, 3, and 4 are firms.

**Example 36**

**Figure 5:**
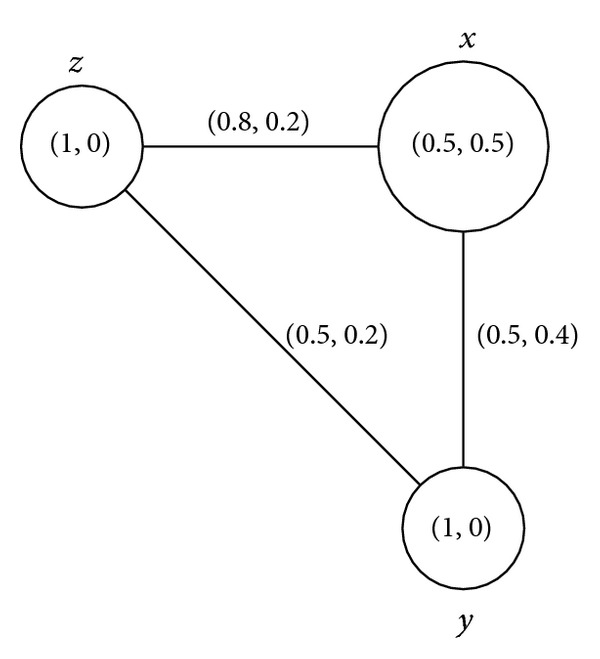
Connected intuitionistic fuzzy graph.

Consider a connected intuitionistic fuzzy graph as shown in Figure 5.

By routine computations, we have *d*(*B*) = (0.5,0.4) and *h*(*B*) = (0.8,0.2). Thus (*s*, *t*)∈(0, *h*(*B*)] means that (*s*, *t*)∈(0,0.8]×(0,0.2]. For 0 < *s* ≤ 0.5 and 0 < *t* ≤ 0.4, *G* ^(*s*,*t*)^ = (*V*, {(*x*, *y*), (*y*, *z*), (*x*, *z*)}). For 0.5 < *s* ≤ 0.8 and 0 < *t* ≤ 0.2, *G* ^(*s*,*t*)^ = (*V*, {(*x*, *z*)}). Thus *G* is a block, an intuitionistic fuzzy block, and full intuitionistic fuzzy block. We note that *G* is not firm.

## 4. Cycles and Trees

**Definition 37**

(1) *G* is called a *cycle* if G* is a cycle.

(2) *G* is called an *intuitionistic fuzzy cycle* if G* is a cycle and there does not exist unique (*x*, *y*) ∈ *E* such that *μ*_*B*_(*x*, *y*) = min⁡{*μ*_*B*_(*u*, *v*) | (*u*, *v*) ∈ *E*}, *ν*_*B*_(*x*, *y*) = max⁡{*ν*_*B*_(*u*, *v*)∣(*u*, *v*) ∈ *E*}.

(3) *G* is called a *weak intuitionistic fuzzy cycle* if there exists (*s*, *t*)∈(0, *h*(*B*)] such that *G* ^(*s*,*t*)^ is a cycle.

(4) *G* is called a *partial intuitionistic fuzzy cycle* if *G* ^(*s*,*t*)^ is a cycle for all (*s*, *t*)∈(*d*(*B*), *h*(*B*)]∪{*h*(*B*)}.

(5) *G* is called a *full intuitionistic fuzzy cycle* if *G* ^(*s*,*t*)^ is a cycle for all (*s*, *t*)∈(0, *h*(*B*)].

**Example 38**

**Figure 6:**
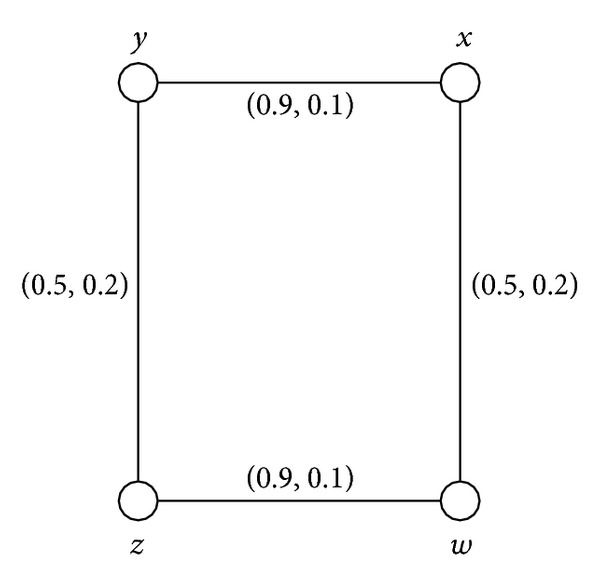
Connected intuitionistic fuzzy graph.

Consider a connected intuitionistic fuzzy graph as shown in Figure 6.

By routine computations, we have *d*(*B*) = (0.5,0.2) and *h*(*B*) = (0.9,0.1). Thus (*s*, *t*)∈(0, *h*(*B*)] means that (*s*, *t*)∈(0,0.9]×(0,0.1]. For 0 < *s* ≤ 0.5 and 0 < *t* ≤ 0.2, *G* ^(*s*,*t*)^ = (*V*, {(*x*, *y*), (*x*, *w*), (*y*, *z*), (*w*, *z*)}). For 0.5 < *s* ≤ 0.9 and 0 < *t* ≤ 0.1, *G* ^(*s*,*t*)^ = (*V*, {(*x*, *y*), (*z*, *w*)}). Thus *G* is an intuitionistic fuzzy cycle and weak intuitionistic fuzzy cycle but *G* is not partial intuitionistic fuzzy cycle.

**Example 39**

**Figure 7:**
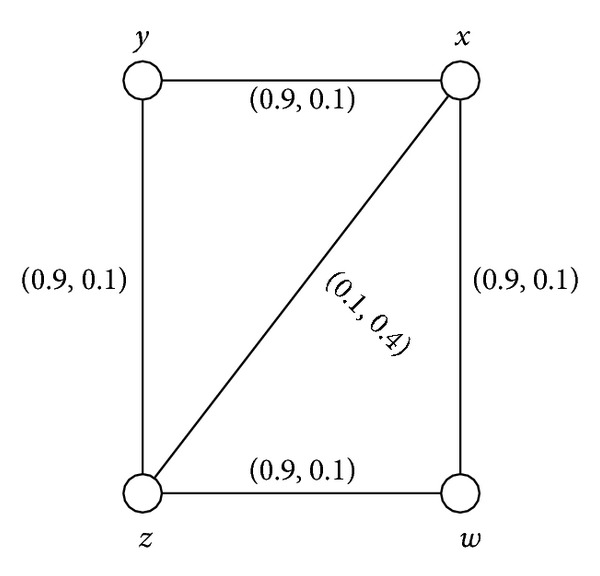
Connected intuitionistic fuzzy graph.

Consider a connected intuitionistic fuzzy graph as shown in Figure 7.

By routine computations, we have *d*(*B*) = (0.1,0.4) and *h*(*B*) = (0.9,0.1). Thus (*s*, *t*)∈(0, *h*(*B*)] means that (*s*, *t*)∈(0,0.9]×(0,0.1]. For 0 < *s* ≤ 0.1 and 0 < *t* ≤ 0.4, *G* ^(*s*,*t*)^ = (*V*, {(*x*, *y*), (*y*, *z*), (*w*, *z*), (*w*, *x*), (*x*, *w*)}) which is not a cycle. For 0.1 < *s* ≤ 0.9 and 0 < *t* ≤ 0.1, *G* ^(*s*,*t*)^ = (*V*, {(*x*, *y*), (*y*, *z*), (*z*, *w*), (*w*, *x*)}) which is a cycle. Thus *G* is not cycle; *G* is a partial intuitionistic fuzzy cycle but not a full intuitionistic fuzzy cycle.

The proofs of the following propositions are trivial.

**Proposition 40**

Suppose that *G* is a cycle. Then *G* is a partial intuitionistic fuzzy cycle if and only if *G* is a full intuitionistic fuzzy cycle.

**Proposition 41**

*G* is a full intuitionistic fuzzy cycle if and only if *G* is a cycle and *B* is constant on *E*.

**Proposition 42**

*G* is a partial intuitionistic fuzzy cycle if and only if *G* ^*h*(*B*)^ is a cycle and | lm (*B*){(0,0)}| ≤ (2,2).

**Proof**

Suppose that *G* is a partial intuitionistic fuzzy cycle. Then clearly *G* ^*h*(*B*)^ is a cycle and in fact *G* ^(*s*,*t*)^ is a cycle for all (*s*, *t*)∈(*d*(*B*), *h*(*B*)]∪{*h*(*B*)}. Suppose that |lm(*B*){(0,0)}| > (2,2). Then ∃(*s*, *t*) such that 0 < *d*(*μ*_*B*_) < *s* < *h*(*μ*_*B*_) and 0 < *d*(*ν*_*B*_) < *t* < *h*(*ν*_*B*_). Hence ∃(*x*, *y*) ∈ *E* such that *μ*_*B*_(*x*, *y*) = *s*, *ν*_*B*_(*x*, *y*) = *t*. Thus (*x*, *y*) ∉ *B* ^*h*(*B*)^ and so *G* ^*h*(*B*)^ is not a cycle, a contradiction.

Conversely, suppose that *G* ^*h*(*B*)^ is a cycle and |lm(*B*){(0,0)}| ≤ (2,2). If |lm(*B*){(0,0)}| = (1,1), then *G* is a full intuitionistic fuzzy cycle by Proposition 41. Suppose that |lm(*B*){(0,0)}| = (2,2). Then lm(*B*){(0,0)} = *d*(*B*), *h*(*B*). Since *G* ^(*s*,*t*)^ = *G* ^*h*(*B*)^ for *d*(*μ*_*B*_) < *s* ≤ *h*(*μ*_*B*_) and *d*(*ν*_*B*_) < *t* ≤ *h*(*ν*_*B*_), it follows that *G* is a partial intuitionistic fuzzy cycle.

**Definition 43**

A connected intuitionistic fuzzy graph *G* = (*A*, *B*) is an intuitionistic fuzzy tree if it has an intuitionistic fuzzy spanning subgraph *H* = (*A*, *C*) which is a tree, where for all arcs (*x*, *y*) not in *H*, *μ*_*B*_(*x*, *y*) < *μ*_*C*_^*∞*^(*x*, *y*), *ν*_*B*_(*x*, *y*) > *ν*_*C*_^*∞*^(*x*, *y*).

**Definition 44**

(1) *G* is called a *forest* if G* is a forest.

(2) *G* is called an intuitionistic fuzzy forest if *G* has an intuitionistic fuzzy spanning subgraph *H* = (*A*, *C*) which is a forest such that, for all (*u*, *v*) ∈ *E* − *W*, *μ*_*B*_(*u*, *v*) < *μ*_*C*_^*∞*^(*u*, *v*) and *ν*_*B*_(*u*, *v*) > *ν*_*C*_^*∞*^(*u*, *v*).

(3) *G* is called a weak intuitionistic fuzzy forest if for all (*s*, *t*)∈(0, *h*(*B*)] such that *G* ^(*s*,*t*)^ is a forest.

(4) *G* is called a partial intuitionistic fuzzy forest if *G* ^(*s*,*t*)^ is a forest for all (*s*, *t*)∈(*d*(*B*), *h*(*B*)]∪{*h*(*B*)}.

(5) *G* is called a full intuitionistic fuzzy forest if *G* ^(*s*,*t*)^ is a forest for all (*s*, *t*)∈(0, *h*(*B*)].

**Example 45**

Consider a connected graph G* = (*V*, *E*) such that *V* = {*x*, *y*, *z*, *w*} and *E* = {(*x*, *y*), (*y*, *z*), (*x*, *w*), (*w*, *z*)}. Let *A* be an intuitionistic fuzzy set of *V* and let *B* be an intuitionistic fuzzy set of *E*⊆*V* × *V* defined by

(17) $$\begin{array}{c} \mu_{A}\left(x\right)=\mu_{A}\left(y\right)=\mu_{A}\left(z\right)=\mu_{A}\left(w\right)=1,\\ \nu_{A}\left(x\right)=\nu_{A}\left(y\right)=\nu_{A}\left(z\right)=\nu_{A}\left(w\right)=0,\\ \mu_{B}\left(x,y\right)=\mu_{B}\left(w,z\right)=0.9,\\ \mu_{B}\left(x,w\right)=\mu_{B}\left(y,z\right)=0.5,\\ \nu_{B}\left(x,y\right)=\nu_{B}\left(w,z\right)=0.1,\\ \nu_{B}\left(x,w\right)=\nu_{B}\left(y,z\right)=0.4. \end{array}$$

By routine computations, we have *d*(*B*) = (0.5,0.4) and *h*(*B*) = (0.9,0.1). For 0 < *s* ≤ 0.5 and 0 < *t* ≤ 0.4, *G* ^(*s*,*t*)^ = (*V*, {(*x*, *w*), (*y*, *z*), (*x*, *y*), (*w*, *z*)}), and for 0.5 < *s* ≤ 0.9 and 0 < *t* ≤ 0.1, *G* ^(*s*,*t*)^ = (*V*, {(*x*, *y*), (*w*, *z*)}). Thus *G* is a partial intuitionistic fuzzy forest but is neither an intuitionistic fuzzy forest nor a full intuitionistic fuzzy forest.

**Proposition 46**

*G* is a full intuitionistic fuzzy forest if and only if *G* is forest.

**Proof**

Suppose that *G* is a full intuitionistic fuzzy forest. Then G* = *G* ^*d*(*B*)^ is a forest.

Conversely, suppose that *G* is a forest. Then G* is a forest and so must be *G* ^(*s*,*t*)^ for all (*s*, *t*)∈(0, *h*(*B*)] since each *G* ^(*s*,*t*)^ is a subgraph of G*. This completes the proof.

**Example 47**

Consider a connected graph G* = (*V*, *E*) such that *V* = {*x*, *y*, *z*} and *E* = {(*x*, *y*), (*y*, *z*), (*x*, *z*)}. Let *A* be an intuitionistic fuzzy set of *V* and let *B* be an intuitionistic fuzzy set of *E*⊆*V* × *V* defined by

(18) $$\begin{array}{c} \mu_{A}\left(x\right)=\mu_{A}\left(y\right)=\mu_{A}\left(z\right)=1,\\ \nu_{A}\left(x\right)=\nu_{A}\left(y\right)=\nu_{A}\left(z\right)=0,\\ \mu_{B}\left(x,y\right)=0.9,\;\;\mu_{B}\left(y,z\right)=0.5,\\ \nu_{B}\left(x,y\right)=0.1,\;\;\nu_{B}\left(y,z\right)=0.4. \end{array}$$

By routine computations, we have *d*(*B*) = (0.5,0.4) and *h*(*B*) = (0.9,0.1). For 0 < *s* ≤ 0.5 and 0 < *t* ≤ 0.4, *G* ^(*s*,*t*)^ = (*V*, {(*x*, *y*), (*y*, *z*)}). For 0.5 < *s* ≤ 0.9 and 0 < *t* ≤ 0.1, *G* ^(*s*,*t*)^ = (*V*, {(*x*, *y*)}). Thus *G* is a forest and a full intuitionistic fuzzy forest without being a constant on *E*. Note that *G* ^*h*^(*B*) has more connected components than G*.

**Proposition 48**

*G* is a weak intuitionistic fuzzy forest if and only if *G* does not contain a cycle whose edges are of strength *h*(*B*).

**Proof**

Suppose that *G* contains a cycle whose edges are of strength *h*(*B*). Then *G* ^(*s*,*t*)^, (*s*, *t*)∈(0, *h*(*B*)], that contains this cycle and so is not a forest. Thus *G* is not a weak intuitionistic fuzzy forest.

Conversely, suppose that *G* does not contain a cycle and all of its edges are of strength *h*(*B*). Then *G* ^*h*(*B*)^ does not contain a cycle and so is a forest.

**Corollary 49**

If *G* is an intuitionistic fuzzy forest, then *G* is a weak intuitionistic fuzzy forest.

**Theorem 50**

*G* is a forest and *B* is constant on *E* if and only if *G* is a full intuitionistic fuzzy forest, G* and *G* ^*h*(*B*)^ have the same number of connected components, and *G* is firm.

**Proof**

Suppose that *G* is a forest and *B* is constant on *E*. Then for all (*s*, *t*)∈(0, *h*(*B*)], *G* ^(*s*,*t*)^ = G* and so *G* is a full intuitionistic fuzzy forest and G* and *G* ^*h*(*B*)^ have the same number of connected components. Clearly, *G* is firm since *B* is a constant on *E*.

Conversely, suppose that *G* is a full intuitionistic fuzzy forest, G* and *G* ^*h*(*B*)^ have the same number of connected components, and *G* is firm. Suppose that ∃(*s*_1_, *t*_1_), (*s*_2_, *t*_2_) ∈ lm(*B*) such that 0 < *s*_1_, *s*_2_, *t*_1_, *t*_2_. Then ∃(*x*, *y*) ∈ *E* such that *μ*_*B*_(*x*, *y*) = *S*_1_ and *ν*_*B*_(*x*, *y*) = *t*_1_. Now (*x*, *y*) ∈ *B* ^(*s*_1_,*t*_1_)^, (*x*, *y*) ∉ *B* ^(*s*_2_,*t*_2_)^. Hence *G* ^(*s*_2_,*t*_2_)^ has more connected components then *G* ^(*s*_1_,*t*_1_)^ since *G* is firm; that is, no vertices were lost. Thus *G* ^*h*(*B*)^ has more connected components than G*, a contradiction.

**Corollary 51**

*G* is a tree and *B* is constant on *E* if and only if *G* is a full intuitionistic fuzzy tree and *G* is firm.

**Definition 52**

(1) *G* is called a *tree* if G* is a tree.

(2) *G* is called an intuitionistic fuzzy tree if *G* has an intuitionistic fuzzy spanning subgraph *H* = (*A*, *C*) which is a tree such that, for all (*u*, *v*) ∈ *E* − *W*, *μ*_*B*_(*u*, *v*) < *μ*_*C*_^*∞*^(*u*, *v*) and *ν*_*B*_(*u*, *v*) > *ν*_*C*_^*∞*^(*u*, *v*).

(3) *G* is called a weak intuitionistic fuzzy tree if for all (*s*, *t*)∈(0, *h*(*B*)] such that *G* ^(*s*,*t*)^ is a tree.

(4) *G* is called a partial intuitionistic fuzzy tree if *G* ^(*s*,*t*)^ is a tree for all (*s*, *t*)∈(*d*(*B*), *h*(*B*)]∪{*h*(*B*)}.

(5) *G* is called a full intuitionistic fuzzy tree if *G* ^(*s*,*t*)^ is a tree for all (*s*, *t*)∈(0, *h*(*B*)].

**Example 53**

Consider a connected graph G* = (*V*, *E*) such that *V* = {*x*, *y*, *z*} and *E* = {(*x*, *y*), (*y*, *z*), (*x*, *z*)}. Let *A* be an intuitionistic fuzzy set of *V* and let *B* be an intuitionistic fuzzy set of *E*⊆*V* × *V* defined by

(19) $$\begin{array}{c} \mu_{A}\left(x\right)=\mu_{A}\left(y\right)=1,\;\;\mu_{A}\left(z\right)=0.5,\\ \nu_{A}\left(x\right)=\nu_{A}\left(y\right)=0,\;\;\nu_{A}\left(z\right)=0.2,\\ \mu_{B}\left(x,y\right)=0.9,\;\;\mu_{B}\left(y,z\right)=0.5,\\ \nu_{B}\left(x,y\right)=0.1,\;\;\nu_{B}\left(y,z\right)=0.4. \end{array}$$

By routine computations, we have *d*(*B*) = (0.5,0.4) and *h*(*B*) = (0.9,0.1). For 0 < *s* ≤ 0.9 and 0 < *t* ≤ 0.1, *G* ^(*s*,*t*)^ = (*V*, {(*x*, *y*), (*y*, *z*)}), and for 0.5 < *s* ≤ 0.9 and 0 < *t* ≤ 0.1, *G* ^(*s*,*t*)^ = (*V*, {(*x*, *y*)}). Thus *G* is a tree, *G* is a full intuitionistic fuzzy tree, and G* and *G* ^*h*^(*B*) have the same number of connected components. However, *G* is not firm and *B* = (*μ*_*B*_, *ν*_*B*_) is not constant on *E*.

**Example 54**

Consider a connected graph G* = (*V*, *E*) such that *V* = {*x*, *y*, *z*} and *E* = {(*x*, *y*), (*y*, *z*), (*x*, *z*)}. Let *A* be an intuitionistic fuzzy set of *V* and let *B* be an intuitionistic fuzzy set of *E*⊆*V* × *V* defined by

(20) $$\begin{array}{c} \mu_{A}\left(x\right)=\mu_{A}\left(y\right)=1,\;\;\mu_{A}\left(z\right)=0.5,\\ \nu_{A}\left(x\right)=\nu_{A}\left(y\right)=0,\;\;\nu_{A}\left(z\right)=0.2,\\ \mu_{B}\left(x,y\right)=0.9,\;\;\mu_{B}\left(x,z\right)=\mu_{B}\left(y,z\right)=0.5,\\ \nu_{B}\left(x,y\right)=0.1,\;\;\nu_{B}\left(x,z\right)=\nu_{B}\left(y,z\right)=0.4. \end{array}$$

By routine computations, we have *d*(*B*) = (0.5,0.4) and *h*(*B*) = (0.9,0.1). For 0 < *s* ≤ 0.5 and 0 < *t* ≤ 0.4, *G* ^(*s*,*t*)^ = (*V*, {(*x*, *y*), (*x*, *z*), (*y*, *z*)}), and for 0.5 < *s* ≤ 0.9 and 0 < *t* ≤ 0.1, *G* ^(*s*,*t*)^ = ({*x*, *y*}, {(*x*, *y*)}). Thus *G* is a partial intuitionistic fuzzy tree but not a full intuitionistic fuzzy tree. *G* is not an intuitionistic fuzzy tree.

We state the following propositions without their proofs.

**Proposition 55**

If *G* is an intuitionistic fuzzy tree, then G is not complete.

**Proposition 56**

If *G* is an intuitionistic fuzzy tree, then arcs of spanning subgraph *H* are the intuitionistic fuzzy bridges of *G*.

**Proposition 57**

If *G* is an intuitionistic fuzzy tree, then internal nodes of spanning subgraph *H* are the intuitionistic fuzzy cut nodes of *G*.

**Proposition 58**

*G* is an intuitionistic fuzzy tree if and only if the following are equivalent:

- (*x*, *y*)* is an intuitionistic fuzzy bridge;*
- *μ*_*B*_^*∞*^(*x*, *y*) = *μ*_*B*_(*x*, *y*)* and ν*_*B*_^*∞*^(*x*, *y*) = *ν*_*B*_(*x*, *y*).

**Proposition 59**

An intuitionistic fuzzy graph is an intuitionistic fuzzy tree if and only if it has a unique maximum spanning tree.

**Definition 60**

For all *s*, *t* ∈ [0,1], one defines $\hat{A^{\left(s,t\right)}}:A^{\left(s,t\right)}\to [0,1]\times [0,1]$ and $\hat{B^{\left(s,t\right)}}:B^{\left(s,t\right)}\to [0,1]\times [0,1]$ by

(21) A(s,t)^(x)=A(x) ∀x∈A(s,t), A(s,t)^(x)=0  otherwise,B(s,t)^(x,y)=B(x,y)∀(x,y)∈B(s,t), B(s,t)^(x,y)=0  otherwise.

**Proposition 61**

Suppose that *G* is firm. If *G* is a weak intuitionistic fuzzy tree, then *G* is an intuitionistic fuzzy tree.

**Proof**

There exist (*s*, *t*)∈(0, *h*(*B*)] such that *G* ^(*s*,*t*)^ is a tree. Since *G* is firm, *G* ^(*s*,*t*)^ is an intuitionistic fuzzy spanning subgraph of *G* which is a tree. If (*u*, *v*) is in *EB* ^(*s*,*t*)^, then *μ*_*B*_(*u*, *v*) < *s*, *ν*_*B*_(*u*, *v*) > *t* and so it follows that *G* is an intuitionistic fuzzy tree.

**Definition 62**

(1) *G* is called connected if G* is connected.

(2) *G* is called intuitionistic fuzzy connected if *G* is intuitionistic fuzzy block.

(3) *G* is called weak intuitionistic fuzzy connected if there exists (*s*, *t*)∈(0, *h*(*B*)] such that *G* ^(*s*,*t*)^ is connected.

(4) *G* is called partial intuitionistic fuzzy connected if *G* ^(*s*,*t*)^ is a connected for for all (*s*, *t*)∈(*d*(*B*), *h*(*B*)]∪{*h*(*B*)}.

(5) *G* is called full intuitionistic fuzzy connected if *G* ^(*s*,*t*)^ is connected for all (*s*, *t*)∈(0, *h*(*B*)].

**Proposition 63**

If *G* is connected, then *G* is weakly connected.

**Proof**

*G* connected implies that G* is connected. Now G* = *G* ^*h*(*B*)^ and so *G* is weakly connected.

**Proposition 64**

If *G* is firm and weakly connected, then *G* is connected.

**Proof**

If *G* ^(*s*,*t*)^ is connected for some (*s*, *t*)∈(0, *h*(*B*)], then G* is connected since *G* is firm.

**Proposition 65**

(1) If *G* is a weak intuitionistic fuzzy tree, then *G* is weakly connected and *G* is a weak intuitionistic fuzzy forest. Conversely, if ∃(*s*_1_, *t*_1_), (*s*_2_, *t*_2_)∈(0, *h*(*B*)] with *s*_1_ < *s*_2_ and *t*_1_ < *t*_2_ such that *G* ^(*s*_1_,*t*_1_)^ is a forest and *G* ^(*s*_2_,*t*_2_)^ is connected, then *G* is a weak intuitionistic fuzzy tree.

(2) *G* is a tree if and only if *G* is a forest and *G* is connected.

(3) *G* is partial intuitionistic fuzzy tree if and only if *G* is a partial intuitionistic fuzzy forest and *G* is partially intuitionistic fuzzy connected.

(4) *G* is a full intuitionistic fuzzy tree if and only if *G* is a full intuitionistic fuzzy forest and *G* is fully connected.

**Proof**

(1) If *G* ^(*s*,*t*)^ is a tree for some (*s*, *t*)∈(0, *h*(*B*)], then *G* ^(*s*,*t*)^ is connected and is a forest. For the converse, we note that *G* ^(*s*_2_,*t*_2_)^ must also be a forest. Since also *G* ^(*s*_2_,*t*_2_)^ is connected, *G* ^(*s*_2_,*t*_2_)^ is a tree.

The proofs of (2), (3), and (4) are immediate.

**Proposition 66**

*G* is firm if and only if *G* ^(*s*,*t*)^ is firm for all (*s*, *t*)∈(0, *h*(*B*)].

**Proof**

Suppose that *G* is firm. Let (*s*, *t*)∈(0, *h*(*B*)]. Let (*x*, *y*) ∈ *μ* ^(*s*,*t*)^. Then

(22) s≤μB(x,y)≤min⁡{μA(x) ∣ x∈V}≤min⁡{μA(x) ∣ x∈μAs},t≥νB(x,y)≥max⁡{νA(x) ∣ x∈V}≥max⁡{νA(x) ∣ x∈νAt}.

Hence max⁡{*μ*_*B*_(*x*, *y*)∣(*x*, *y*) ∈ *μ*_*B*_^*s*^} ≤ min⁡{*μ*_*A*_(*x*) | *x* ∈ *μ*_*A*_^*s*^} and min⁡{*ν*_*B*_(*x*, *y*)∣(*x*, *y*) ∈ *ν*_*B*_^*t*^} ≥ max⁡{*ν*_*A*_(*x*) | *x* ∈ *ν*_*A*_^*t*^}. Thus we conclude that *B* ^(*s*,*t*)∗^ = *B* ^(*s*,*t*)^ and *A* ^(*s*,*t*)∗^ = *A* ^(*s*,*t*)^ and *G* ^(*s*,*t*)^ is firm.

Conversely, suppose that *G* ^(*s*,*t*)^ is firm for all (*s*, *t*)∈(0, *h*(*B*)]. Let min⁡{*μ*_*A*_(*x*) | *x* ∈ *V*} = *s*_0_, and let max⁡{*ν*_*A*_(*x*) | *x* ∈ *V*} = *t*_0_. Then *t*_0_ > 0. Now max⁡{*μ*_*B*_(*x*, *y*) | (*x*, *y*) ∈ *μ*_*B*_^*s*_0_^} ≤ *s*_0_ and min⁡{*ν*_*B*_(*x*, *y*)∣(*x*, *y*) ∈ *ν*_*B*_^*t*_0_^} ≥ *t*_0_ since *G* ^(*s*_0_,*t*_0_)^ is firm and *V* = *A* ^(*s*_0_,*t*_0_)^ = *A* ^(*s*_0_,*t*_0_)∗^. Let (*x*, *y*) ∈ *E* − *B* ^(*s*,*t*)∗^. Then *μ*_*B*_(*x*, *y*) < *s*_0_, *ν*_*B*_(*x*, *y*) > *t*_0_. Thus

(23) max⁡{μB(x,y) ∣ (x,y)∈E}≤s0=min⁡{μA(x) ∣ x∈V},min⁡{νB(x,y) ∣ (x,y)∈E}≥t0=max⁡{νA(x) ∣ x∈V}.

Hence *G* is firm.

## 5. Conclusions

In a network, each arc is assigned a weight. The weight of a path or a cycle is defined as the minimum weight of its arcs. The maximum of weights of all paths between two nodes is defined as the strength of connectedness between the nodes. In network applications, the reduction in the strength of connectedness is more relevant than the total disconnection of the graph. A graph is totally weighted if both node set and arc set are weighted. Fuzzy graph theory is finding an increasing number of applications in modeling real time systems. Since intuitionistic fuzzy models give more precision, flexibility, and compatibility to the system as compared to the fuzzy models, we have investigated some properties of intuitionistic fuzzy cycles, intuitionistic fuzzy trees, intuitionistic fuzzy bridges, and intuitionistic fuzzy cut vertices in intuitionistic fuzzy graphs in this paper. We plan to extend our research of fuzzification to (1) bipolar fuzzy trees, (2) soft cycles and soft trees, (3), and rough cycles and rough trees.
